# Priorities to Promote Participant Engagement in the Participant Engagement and Cancer Genome Sequencing (PE-CGS) Network

**DOI:** 10.1158/1055-9965.EPI-22-0356

**Published:** 2023-02-15

**Authors:** Anne LR. Schuster, Norah L. Crossnohere, Melinda Bachini, Cindy K. Blair, John D. Carpten, Elizabeth B. Claus, Graham A. Colditz, Li Ding, Bettina F. Drake, Ryan C. Fields, Katherine A. Janeway, Bethany M. Kwan, Heinz-Josef Lenz, Qin Ma, Shiraz I. Mishra, Electra D. Paskett, Timothy R. Rebbeck, Charité Ricker, Mariana C. Stern, Andrew L. Sussman, Jessica C. Tiner, Jeffrey M. Trent, Roel GW. Verhaak, Nikhil Wagle, Cheryl Willman, John FP. Bridges, for the Participant Engagement and Cancer Genome Sequencing (PE-CGS) Network

**Affiliations:** 1 Department of Biomedical Informatics, The Ohio State University College of Medicine, Columbus, Ohio.; 2 Division of General Internal Medicine, Department of Internal Medicine, The Ohio State University, Columbus, Ohio.; 3 Cholangiocarcinoma Foundation, Herriman, Utah.; 4 Department of Internal Medicine, University of New Mexico Comprehensive Cancer Center and Health Sciences Center, Albuquerque, New Mexico.; 5 Institute of Translational Genomics, Department of Translational Genomics, Keck School of Medicine USC, Norris Comprehensive Cancer Center, Los Angeles, California.; 6 Department of Biostatistics, Yale School of Public Health, New Haven, Connecticut.; 7 Department of Neurosurgery, Brigham and Women's Hospital, Boston, Massachusetts.; 8 Department of Surgery, Washington University School of Medicine, Alvin J. Siteman Cancer Center, St. Louis, Missouri.; 9 Division of Oncology, Department of Medicine, Washington University School of Medicine, St. Louis, Missouri.; 10 Division of Public Health Sciences, Washington University School of Medicine, Alvin J. Siteman Cancer Center, St. Louis, Missouri.; 11 Division of General Surgery, Washington University School of Medicine, Alvin J. Siteman Cancer Center, St. Louis, Missouri.; 12 Dana-Farber / Boston Children's Cancer and Blood Disorders Center, and Broad Institute of MIT and Harvard, Harvard Medical School, Boston, Massachusetts.; 13 Department of Emergency Medicine, University of Colorado Anschutz Medical Campus, Aurora, Colorado.; 14 Keck School of Medicine of USC, Norris Comprehensive Cancer Center, Los Angeles, California.; 15 Departments of Pediatrics and Family and Community Medicine, University of New Mexico Comprehensive Cancer Center, University of New Mexico Health Sciences Center, Albuquerque, New Mexico.; 16 Division of Cancer Prevention and Control, Department of Internal Medicine, College of Medicine; Division of Epidemiology, College of Public Health, The Ohio State University, Columbus, Ohio.; 17 Harvard TH Chan School of Public Health, Broad Institute of MIT and Harvard, and the Dana-Farber Cancer Institute, Boston, Massachusetts.; 18 Division of Medical Oncology, Department of Medicine, Keck School of Medicine USC, Norris Comprehensive Cancer Center, Los Angeles, California.; 19 Department of Population and Public Health Sciences & Urology, Keck School of Medicine of USC, Norris Comprehensive Cancer Center, Los Angeles, California.; 20 Department of Family and Community Medicine, University of New Mexico Comprehensive Cancer Center and Health Sciences Center, Albuquerque, New Mexico.; 21 Epidemiology and Genomics Research Program, Division of Cancer Control and Population Sciences, National Cancer Institute, Bethesda, Maryland.; 22 Translational Genomics Research Institute part of City of Hope, Phoenix, Arizona.; 23 The Jackson Laboratory for Genomic Medicine, Farmington, Connecticut.; 24 Dana-Farber Cancer Institute, Broad Institute of MIT and Harvard, Harvard Medical School, Dana-Farber/Harvard Cancer Center, and Count Me In, Boston, Massachusetts.; 25 Department of Laboratory Medicine and Pathology, Mayo Clinic Comprehensive Cancer Center, Mayo Clinic, Rochester, Minnesota.; 26 University of New Mexico School of Medicine and Comprehensive Cancer Center, Albuquerque, New Mexico.

## Abstract

**Background:**

Engaging diverse populations in cancer genomics research is of critical importance and is a fundamental goal of the NCI Participant Engagement and Cancer Genome Sequencing (PE-CGS) Network. Established as part of the Cancer Moonshot, PE-CGS is a consortium of stakeholders including clinicians, scientists, genetic counselors, and representatives of potential study participants and their communities. Participant engagement is an ongoing, bidirectional, and mutually beneficial interaction between study participants and researchers. PE-CGS sought to set priorities in participant engagement for conducting the network's research.

**Methods:**

PE-CGS deliberatively engaged its stakeholders in the following four-phase process to set the network's research priorities in participant engagement: (i) a brainstorming exercise to elicit potential priorities; (ii) a 2-day virtual meeting to discuss priorities; (iii) recommendations from the PE-CGS External Advisory Panel to refine priorities; and (iv) a virtual meeting to set priorities.

**Results:**

Nearly 150 PE-CGS stakeholders engaged in the process. Five priorities were set: (i) tailor education and communication materials for participants throughout the research process; (ii) identify measures of participant engagement; (iii) identify optimal participant engagement strategies; (iv) understand cancer disparities in the context of cancer genomics research; and (v) personalize the return of genomics findings to participants.

**Conclusions:**

PE-CGS is pursuing these priorities to meaningfully engage diverse and underrepresented patients with cancer and posttreatment cancer survivors as participants in cancer genomics research and, subsequently, generate new discoveries.

**Impact:**

Data from PE-CGS will be shared with the broader scientific community in a manner consistent with participant informed consent and community agreement.

## Introduction

Landmark cancer genome sequencing programs such as The Cancer Genome Atlas have deepened our understanding of cancer biology and generated opportunities to develop new cancer therapies, diagnostic methods, and preventive strategies (1, 2). There remain, however, significant gaps in our understanding of the relationship between cancer and genetics. First, hundreds of cancer subtypes have not been sufficiently characterized (3). Second, findings from cancer genome sequencing studies are not generalizable to the entire population because of the inequitable participation of people from racial and ethnic minority groups and from adolescents and young adults (4). Studies find that less than 0.5% of tumors sequenced in national genomic initiatives were from people of American Indian, Alaska Native, Native Hawaiian, or Pacific Islander descent, combined (5, 6). Small percentages of Black and Hispanic patients have been represented in these efforts, which is a concern given their increased burden of most cancers (6, 7). Many of these studies prioritized convenience of sample availability and access over representation, even for tumor types that represent significant disparities within certain populations. Finally, many cancer genomic studies lack adequate clinical and epidemiologic data, which limits inferences and the translational impact of sequencing data (8).

Participant engagement in research is one approach to address these gaps. Participant engagement is a key focus of the recently established Participant Engagement and Cancer Genome Sequencing (PE-CGS) Network (pe-cgs.org). The PE-CGS Network was established by the NCI as part of the Cancer Moonshot (9). Participant engagement, a foundational principle of the PE-CGS Network, is defined as an ongoing, bidirectional, and mutually beneficial interaction among study participants and researchers, in which participants are included as an integral part throughout the research processes (3, 10). Participant engagement is related to but distinct from similar concepts such as patient or stakeholder engagement in research, which refers to relationships with people who may or may not be formally participating in an active research initiative. The PE-CGS Network promotes and supports the direct engagement of diverse and underrepresented patients with cancer and posttreatment cancer survivors as participants and partners in planning, conduct, and dissemination of cancer genomics research. The five NCI-funded PE-CGS Network centers are conducting rigorous cancer genome sequencing studies to address research gaps in the molecular profiles of multiple cancer types. These molecular profiles include highly lethal cancers; rare cancers or subsets of rare cancers; cancers with an early age of onset; cancers in understudied populations; and cancers with high disparities in mortality and/or incidence.

The need for participant engagement in cancer genomics research, however, has outpaced research on optimal strategies of engagement. The PE-CGS Network aims to determine best practices for participant engagement strategies for aspects such as: directly reaching and communicating with potential participants about the goals and values of genomic characterization; facilitating the gathering of reliable and high-quality information from participants; and effectively communicating and disseminating research results to study participants (3). A recent paper by Rebbeck and colleagues presents a novel framework that could guide investigators and communities interested in optimizing the methods of participant engagement by working in partnership, supporting representation in research, and facilitating the rigorous conduct of genomics research (11). This framework considers how engagement can ensure the use of robust methods for a range of study activities including recruitment, retention, return of genomic results, quality of engagement, and follow-up. It highlights several issues pertinent to promoting participant engagement in the PE-CGS Network. The PE-CGS Network's challenge was identifying where to focus its efforts to support its long-term goal of determining best practices for participant engagement.

The PE-CGS Network sought to set priorities in participant engagement for the Network's research and to set them early in the Network's formation as a principle of transparency and accountability. This article describes the process and results of deliberatively engaging PE-CGS stakeholders to set the network's research priorities in participant engagement. The priorities are likely to be of interest to funders, scientists, and other networks embarking on participant engagement in cancer genomics research. Yet, we also aim to highlight the scientific value and resources associated with addressing the network's priorities. Addressing these priorities position the PE-CGS Network to overcome the inequitable representation of a range of diverse populations in cancer genomics research and generate unique data that can address critical gaps in knowledge. The data generated by the PE-CGS Network will be a valuable resource made available to the broader scientific community in a manner consistent with participant informed consent and in compliance with the Cancer Moonshot Public Access and Data Sharing Policy (12).

## Materials and Methods

A four-phase deliberative engagement process was conducted in the fall of 2021 to identify priorities in participant engagement for the PE-CGS Network. Setting research priorities can provide direction and consensus about areas of importance to stakeholders and where increased research effort will make a significant impact on knowledge or practice (13, 14). It also helps ensure that research makes efficient and equitable use of resources while reducing duplicative efforts (15).

### Consortium stakeholders

The PE-CGS Network is a consortium of stakeholders selected through a competitive, peer-reviewed grant application process that includes geneticists, oncologists, epidemiologists, physician scientists, genetic counselors, computational biologists, behavioral researchers, genomic scientists, cancer cell biologists, scientists on the ethical, legal, and social implications of genetics and genomics, and representatives of potential study participants and their communities. Stakeholders were invited to contribute to the deliberative engagement process as scientists if they were affiliated with the PE-CGS Network as of October 2021. The Network is funded by the NCI and currently comprised of five Research Centers, one Coordinating Center, and an External Advisory Panel.

The Research Centers will collectively address gaps in knowledge about the molecular characterization of several types of cancers. In addition to collecting biospecimens, the Research Centers plan to collect detailed and comprehensive data on clinical, epidemiologic, behavioral, and/or psychosocial factors including from medical records and patient surveys and/or interviews. The Research Centers will focus on diverse patient populations with different cancers, at different stages of the life course, and representing different racial/ethnic identities (Table 1). The Research Centers include: Center for Optimization of Participant Engagement for Cancer Characterization (COPECC; University of Southern California); Count Me In PE-CGS Center (Broad Institute, Dana-Farber Cancer Institute, Boston Children's Hospital); Engagement of American Indians of Southwestern Tribal Nations in Cancer Genome Sequencing (University of New Mexico, Translational Genomics Research Institute; part of City of Hope, Black Hills Center for American Indian Health); OPTimIzing engagement in the discovery of molecular evolution of low-grade glioma (OPTIMUM; Yale University, University of Colorado, The Jackson Laboratory for Genomic Medicine, Brigham and Women's Hospital, Beth Israel Deaconess Medical Center); and Washington University Participant Engagement and Cancer Genomic Sequencing Center (WU-PE-CGS; Washington University in St. Louis). The Coordinating Center is at The Ohio State University. The PE-CGS Network also has a 6-member External Advisory Panel that provides input on scientific direction, evaluates the progress of the network, and offers recommendations for improvement. External Advisory Panel members (see Acknowledgments) include individuals with a history of cancer, cancer patient advocates, medical and precision oncologists, and scientists with expertise in genetic epidemiology, genomics and systems biology.

**Table 1.** Research centers in the PE-CGS network.

			Knowledge gaps				
Research center	Cancer focus	Population focus	Rare cancer	Highly lethal	Early onset	Disparities	Understudied population
**COPECC**, University of Southern California	Colorectal cancer	Hispanics/Latinos			**X**	**X**	**X**
**Count Me In PE-CGS Center** Broad Institute, Dana-Farber Cancer Institute, Boston Children's Hospital	Osteosarcoma Leiomyosarcoma	Children with osteosarcoma, adults with leiomyosarcoma	**X**		**X**		**X**
**Engagement of American Indians of Southwestern Tribal Nations in Cancer Genome Sequencing** University of New Mexico, the Mayo Clinic, the Translational Genomics Research Institute (TGen), Black Hills Center for American Indian Health	Disparities cancers (gastrointestinal, hepatobiliary, genitourinary, and hormone dependent)	American Indians of Southwestern Tribal Nations				**X**	**X**
**OPTIMUM**, Yale University, University of Colorado, Jackson Laboratory for Genomic Medicine, Brigham and Women's Hospital, and Beth Israel Deaconess Medical Center	Low-grade glioma	Young to middle-aged adults	**X**	**X**	**X**		**X**
**WU-PE-CGS**, Washington University in St. Louis	Cholangiocarcinoma Colorectal cancer Multiple myeloma	Adults with cholangiocarcinoma, Black Americans under age 50 with colorectal cancer, Black Americans with multiple myeloma	**X**	**X**	**X**	**X**	**X**

**Table 2.** Priorities for the PE-CGS network.

Priority	Brief description	Example^a^ from PE-CGS research centers
1. Tailor education and communication materials for participants	Develop participant-centric communication and educational materials that span research process	*Promote improved messaging and education*: The OPTIMUM Research Center plans to support bidirectional and patient-centric messaging and education through the use of a novel digital tool called Hugo Health Platform.
2. Identify measures of participant engagement	Identify, adapt, or develop measures of engagement that are meaningful to participants	*Integrate patient input in data collection*: The Count Me In PE-CGS Research Center used an iterative feedback-loop to gather community input on all aspects of study design, including their planned patient intake survey.
3. Identify optimal strategies for participant engagement	Identify comparatively more effective participant engagement strategies via conduct of rigorous research	*Understand refusal to participate in research*: The WU-PE-CGS Research Center plans to engage people who decline to participate for insight into how refusal reflects values and/or rectifiable factors, such as trust, study design, or recruitment strategies
4. Understand cancer disparities in context of genomics research	Study role that biology plays in cancer disparities in concert with clinical, epidemiologic, and social determinants of health	*Collect and integrate data on environmental exposures:* The Research Center called “Engagement of American Indians of Southwestern Tribal Nations in Cancer Genome Sequencing” will evaluate the association between cancer and environmental exposures resulting from abandoned hard rock and uranium mines
5. Personalize return of genomics findings to participants	Identify how germline and somatic results are being framed and how they account for participant preferences	*Preferences for delivery of genomics results:* The COPECC Research Center will assess participants’ preferences for who should deliver genomic testing results to participants in addition to understanding what results to return.

^a^One Research Center from the PE-CGS Network was selected to exemplify each priority, but the activity described in the example may not be unique to that particular Research Center and may apply to additional Research Centers in the PE-CGS Network.

### Setting priorities

**Figure 1.**
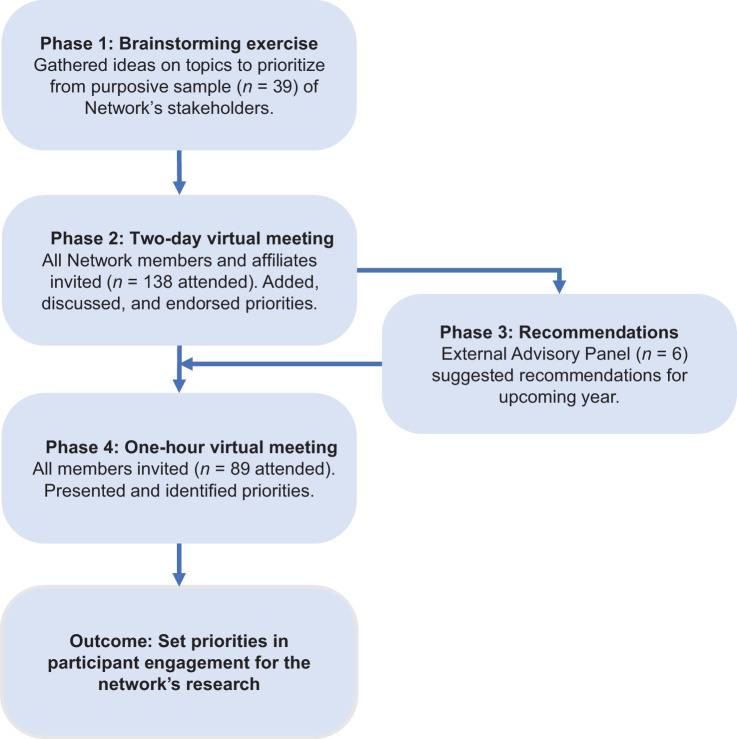
The four-phase process to identify Network priorities. Depicts the stages of the process used to identify Network priorities.

Figure 1 depicts the four-phase deliberative engagement process that was conducted to set priorities in participant engagement for the PE-CGS Network. The process comprised: (i) a brainstorming exercise to elicit potential priorities; (ii) a 2-day virtual meeting to add, discuss, and endorse priorities; (iii) recommendations from the PE-CGS External Advisory Panel to refine priorities; and (iv) a 1-hour virtual meeting to discuss and set priorities.

In phase one, 39 stakeholders who were scheduled to speak at the PE-CGS Network's 2-day virtual meeting (phase two) completed a brief online survey. The speakers represented stakeholders across several roles including: oncologists, epidemiologists, patients with cancer, computational biologists, cancer cell biologists, genetic counselors, physician scientists, behavioral researchers, representatives of potential study participants and members of their communities. The survey asked two open-ended questions to gather ideas for further discussion and prioritization during the virtual meeting, including: (i) “What are a few topics that you think the PE-CGS Network should collaborate on?” and (ii) “What ideas do you have for activities that could foster collaboration across the PE-CGS Network?.” We screened responses to remove duplicates and, where necessary, rephrased responses to improve readability.

In phase two, the PE-CGS Network hosted a 2-day virtual meeting on November 1 and 2, 2021. This was the first annual meeting as a fully formed Network. Stakeholders from all roles were represented at this meeting. The objectives of the meeting were to articulate the Network's goals and vision; understand the science of each Research Center; learn from participant representatives; and identify areas of collaboration for the upcoming year. The meeting included both scientific presentations and moderated discussion. Attendees were invited to use the meeting's chat function to ask questions and make comments. There were also opportunities for stakeholders to share and discuss their ideas. A live captioner transcribed the meeting.

**Figure 2.**
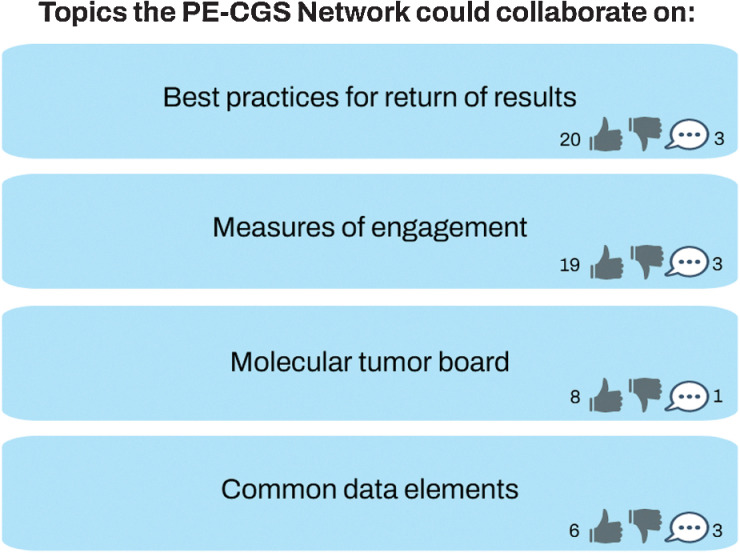
Example of online platform for real-time brainstorming and voting. Contains a modified display of comments and voting on online platform (https://reetro.io).

Throughout the meeting, stakeholders were asked to use the online platform Reetro (https://reetro.io) for real-time brainstorming and interaction (Fig. 2). Reetro is designed to collect participant thoughts, ideas, and feedback as comments. Updates are made in real-time so everything is instantly visible to everyone on the platform. Attendees could add new comments or reply to comments and endorse ideas by giving a “thumbs up” to comments that they thought should be a higher priority. In addition, attendees could endorse ideas verbally or via the chat. We pre-populated the fields on the online platform with responses gathered from the survey results in phase one. After the meeting, responses to the Reetro and comments identified in the transcript or chat log were grouped into categories that reflected distinct priorities. The number of thumbs up that each category received were tallied and used to rank the categories. One author (A.L.R. Schuster) reviewed the transcripts from the meeting to further identify representative examples of the respective categories.

In phase three, members of the External Advisory Panel met to summarize the strengths of the Network and generate a list of recommendations for improvement. As noted above, the External Advisory Panel includes individuals with a history of cancer, cancer patient advocates, oncologists, and scientists. The External Advisory Panel based its summary and recommendations on the material presented and discussed during the 2-day virtual meeting. In particular, members of the External Advisory Panel were asked to highlight the strengths of the Network; and offer recommendations for improvement. After the 2-day virtual meeting, the External Advisory Panel members met with each other to discuss these questions, and specifically to: discuss, agree upon, and record their recommendations. These recommendations were provided in verbal and written-formats to the PE-CGS Network's Steering Committee. We compared the External Advisory Panel's list of written recommendations to the categories and examples identified in phase two. Two authors (A.L.R. Schuster, N.L. Crossnohere) incorporated issues and examples identified by the External Advisory Panel into the descriptions of the priorities identified in phase two.

In phase four, the results of phases one through three were presented during a 1-hour virtual meeting that was open to all members of the Network. Stakeholders from all roles within the PE-CGS Network were represented at this meeting. This meeting was held on November 18, 2021, approximately two weeks after the 2-day virtual meeting. Discussions during this presentation were used to clarify how the categories were ranked as well as the relationships between the categories. The final setting of the priorities was largely based on their urgency of facilitating immediate next steps of the Research Centers in the PE-CGS Network.

### Data availability

The data generated in this study are available upon request from the corresponding author.

## Results

In total, 138 stakeholders affiliated with the PE-CGS Network contributed to at least one phase of the process. Many of the stakeholders were involved because of their role as scientists, clinicians, genetic counselors, or research funders (93%). Community and cancer patient representatives were also key stakeholders engaged in the process (7%).

### Priorities for participant engagement in the PE-CGS network

Eighteen potential priorities were identified during the brainstorming exercise of phase one of the deliberative engagement process. They were thematically grouped into the following 10 distinct categories after phase two of the process: (i) optimization of engagement strategies; (ii) diversity and cancer disparities; (iii) return of genomic sequencing results; (iv) education and communication materials; (v) measures of participant engagement; (vi) genome sequencing protocol standardization; (vii) ethical, legal, and social issues; (viii) collection and integration of data from multiple sources (ix); standardization and harmonization of data; and (x) translation of genomic discovery. The first five of these categories were collectively endorsed by more stakeholders than the last five. From these categories, five PE-CGS Network priorities for participant engagement were identified and set throughout the process (Table 2).

### Priority 1: tailoring education and communication materials for participants

Participant-centric communication and educational materials include recruitment materials; consent documents; permission for data use; statements accompanying genomic findings; and dissemination of genomic results and aggregate study findings. To tailor this material for participants, the Network should use best practices for communicating with the general public. Best practices include writing at recommended reading levels; adapting the language used to be sensitive to the cultural context; translating material into multiple languages; and, using non-text content such as pictures, images, and figures.

Stakeholders paid particular attention to considering how educational materials could adequately support informed consent. Some use in-person consent formats and others obtain consent via technology. In addition to considering this range of formats, stakeholders also wanted to consider the public's generally lower levels of knowledge about cancer genomics, cancer genomics research, and genomic testing—both germline and somatic. Stakeholders also identified gaps in knowledge about how best to communicate individual genomic results and aggregate study results, especially for diverse populations. When communicating genomic results, they understand that it is critical to be culturally adaptive and mindful of differences in language and literacy-levels (16).

### Priority 2: identifying measures of engagement

Identifying, adapting, and developing measures of engagement was the second-highest priority. There is a lack of evidence about the impact of participant engagement in cancer genomics research (17). A variety of process and outcome measures were proposed to evaluate the impact of participant engagement. These measures included participants’ satisfaction with the process; participants’ changes in knowledge; studies’ enrollment and retention rates; changes in trust between researchers and participants; and genomic and clinical discoveries.

Stakeholders noted that measures of engagement needed to map back to how participant engagement was defined and who was engaged. They recommended that the Network explicitly extend its definition of engagement beyond enrollment, retention, and return of results. Stakeholders shared that existing measures of engagement may not address the needs of underserved or underrepresented populations being recruited in the PE-CGS Network. As a result, it was noted that the Network may need to adapt existing measures or to develop new measures to align with participants’ preferences. Stakeholders commented that the Network would likely need to apply mixed methods research to examine whether existing measures are appropriate, how existing measures could be adapted, and/or how new measures could be developed.

### Priority 3: identifying optimal engagement strategies

Stakeholders noted that we lack evidence on the effectiveness of different participant engagement strategies, especially their use in underserved and historically underrepresented populations. The stakeholders discussed the need to identify and implement appropriate research designs to test engagement strategies and detect when an engagement strategy is not working and a new strategy should be tested. For instance, rapid-cycle research (18, 19) was discussed as one approach to iteratively identify and resolve sub-optimal strategies. Another approach discussed was using comparative effectiveness research to test culturally tailored engagement strategies against standard-of-care strategies and regularly evaluating the two with preestablished thresholds. Unmet thresholds would then trigger a change in strategies.

Stakeholders discussed the opportunity to improve engagement strategies by learning from people who choose to not participate in a study and studying why they decline to participate. This is important because access to research participation and the generation of research findings that reflect the diversity of the U.S. population are matters of justice. Similarly, stakeholders said it was important to learn from those who drop out of the study. Studying the reasons for study decline and study dropout will be challenging.

### Priority 4: understanding the role of cancer disparities in cancer genomics

Stakeholders emphasized the importance of this research priority to ensure that participants and communities benefit equitably from participating in cancer genomics research. Stakeholders identified the importance of participation of various underserved populations in the Network's studies, including participants across ages, gender identities, racial and ethnic groups, and rural and urban residence. Doing so would help ensure that the samples studied are representative of diverse populations and lead to results that would be generalizable to those populations.

Stakeholders also endorsed the importance of collecting data on the social determinants of health, such as environment, socioeconomic status, and structural racism. The Network could then integrate the data on the social determinants of health (20) with cancer genomics data. Doing so was seen as vital for enhancing our understanding of cancer disparities and promoting health equity. In addition, stakeholders advocated for seeking input from communities about social determinants of health data they would like to see collected. This would not only help ensure the study could accrue benefit to communities, but would also allow communities to plan for implications of possible study findings.

### Priority 5: personalizing return of genomics findings

Personalizing the return of results addressed several issues that are sensitive to participant preferences, especially in light of existing gaps in knowledge. A number of issues were noted related to the return of germline and/or somatic results. For instance, citing uncertainty about clinical actionability related to returning somatic results (21–24), stakeholders recommended ascertaining participants’ preferences for the types of results they would like to receive. Returning results of genomics findings is complicated in understudied populations in which reference genomes do not exist and variants of uncertain significance may be common (11), which influences the need to gather participants’ preferences. Stakeholders questioned how best to ascertain participants’ preferences, when to ascertain them, and if and how to revisit their preferences over time.

Given uncertainty about how best to communicate results, stakeholders also recommended seeking participants’ input on how to deliver results and to aid their understanding of results, especially given that the results may not be actionable. It was suggested that it could be beneficial to understand if participants prefer to hear the results from a doctor, a nurse, a genetic counselor, or a study member. Stakeholders additionally raised a point about providing informational materials to help empower the affected patient and facilitate communication between the participant and their family members regarding the genomic results and potential risks (or not) for their relatives. This would necessitate taking into consideration legal issues around returning germline results to family members who could also be affected. Stakeholders noted the value of the Network identifying best practices on these issues.

## Discussion

Establishing the priorities for any Network is an important early step in developing a shared vision and goals across diverse teams of researchers and cancer populations. Through a highly interactive process drawing upon the expertise of scientists, clinicians, community representatives, patients living with cancer, and survivors of cancer, we set the PE-CGS Network's priority areas for enhancing participant engagement. The highest priorities for this field include tailoring education and communication materials for participants and identifying measures of participant engagement. The priorities that we identified are complementary to the overarching goals of the PE-CGS Network, which are to determine best practices for participant engagement in cancer genomics research and address research gaps in molecular profiles of cancer.

The PE-CGS Network has established three cross-network subcommittees focused on: (i) health equity (ii), participant engagement, and (iii) return of results. These subcommittees are currently developing approaches and resources to address these priorities. For instance, Network members recently published a novel framework to optimize equitable representation in genomics research (11). This framework touches on priority 1, to tailor communication and education to participants, as well as priority 4, to understand the role of disparities in cancer genomics. Other resources that are currently in development to meet the priorities of PE-CGS will broadly address existing gaps in knowledge around topics such as best practices for return of results, definitions of engagement (25); measures of participant engagement in cancer genomics research, a toolbox for optimal engagement strategies, and harmonized measures related to the social determinants of health to foster a deeper understanding of cancer disparities.

As resources become available, they will be easily accessible through the Network's website (pe-cgs.org) and actively disseminated through additional communication channels, including social media. The types of information, resources, and materials provided on the PE-CGS website will be informed by the practices of established and long-standing transdisciplinary networks such as the eMERGE Network (https://emerge-network.org/) and the CSER Consortium (https://cser-consortium.org/). For instance, the eMERGE Network features a dashboard that summarizes key metrics about the Network including the number of Network publications and the number of Network cohort participants. The CSER Consortium provides resources and research materials on their website which include harmonized measures and consent forms and educational materials in English and Spanish.

The likely impact of the PE-CGS Network extends beyond these resources. In compliance with the Beau Biden Cancer Moonshot Open Access and Data Sharing Policy (12), the PE-CGS Network will: make its publications available to the public; and, to the extent possible, the underlying primary data behind them through appropriate data repositories and in a manner consistent with participant informed consent and community agreement. As an example, the PE-CGS Network's Research Centers will securely store, prepare, and transmit genomic data to the NCI Genomic Data Commons for subsequent sharing with the broader scientific community in a manner consistent with participant informed consent, as well as Tribal agreement in the specific case of the PE-CGS Research Center on Engagement of American Indians of Southwestern Tribal Nations in Cancer Genome Sequencing. The sharing of PE-CGS Network publications and underlying data are critical mechanisms to accelerate cancer research in understudied areas of cancer genomics.

Despite some progress, there is still much work to be done to improve racial and ethnic diversity in cancer genomics research and in cancer disparities more generally. There are significant gaps in cancer research that will exacerbate cancer disparities for Black, Hispanic, Indigenous, and additional populations underrepresented in cancer research such as adolescents and young adults (4, 5). Cancer genome sequencing research represents a tremendous opportunity to ultimately deliver personalized, targeted cancer care. However, many people in underrepresented populations have been historically excluded from genomic studies, and thus do not benefit from these personalized approaches. Moreover, the individual- and community-level implications of cancer genomics research raise important questions for participant and community engagement around consent, biospecimen use, data privacy and protections, data sharing, and the description of findings that are not unconsciously biased. The PE-CGS Network is poised to study these dynamic relationships between participant and community engagement and answer questions about the specificity versus generalizability of strategies to engage diverse populations.

Meaningful coordination and cooperation will be needed to address the PE-CGS Network's priorities. There are several strategies that may help. These include establishing subcommittees; holding Network-wide “All Hands” meetings; and securely sharing data and materials. Collectively, these strategies will promote the development of shared mental models (26) to ensure that there is common understanding of concepts, constructs, and terminology and enable working effectively and cohesively across the Network. Moreover, these strategies will facilitate cross-talk, problem solving, and collaboration to generate standardized and harmonized data as well as scientific products such as publications, white papers, and toolkits. In addition, there is an opportunity to leverage the Network's infrastructure to build diverse cohorts via open consent processes across some/all Centers or more expansive outreach efforts.

The priorities we identified will only be realized though transdisciplinary collaboration. The PE-CGS Network is comprised of transdisciplinary researchers and is also interested in forming strategic partnerships with networks and researchers external to PE-CGS. Several priorities endorsed by our Network were designed to provide opportunities for members in the PE-CGS Network to innovate and cocreate by bringing together individuals with relevant and unique perspectives and expertise.

The prioritization process presented here is not without limitations. First, our process relied on engagement and results are not necessarily generalizable to other research networks. However, we believe the findings may be of interest to other researchers and national Networks as a guide or reference for issues to consider. Participating stakeholders are members of the PE-CGS Network and were selected through a competitive process. They represent the relevant range of transdisciplinary academic stakeholders and members of the lay community to achieve the Network's goals and priorities. The percentage of community and cancer patient representatives was relatively low, but these were individuals highly attuned to their communities’ needs and preferences (11, 14), and versed in research to meaningfully participate in a research priority setting process. As the PE-CGS Network continues to mature, it will continually strive to meaningfully include participant representatives throughout its work. This is increasingly important. Many of the research priorities we identify, including identifying measures of engagement and identifying effective engagement strategies, overlap several upcoming funding opportunities announced by the Patient Centered Outcomes Research Institute (27). Similarly, our research priorities address several central pillars of the recently reignited Cancer Moonshot, including learning more from patients with cancer (28, 29).

It is also possible that the transparent nature of the process, though necessary for engagement, may have resulted in yea-saying. Another limitation pertains to the COVID-19 pandemic, which prevented us from conducting the meetings in person. It is unclear if or how this may have changed the priorities we identified. An unforeseen benefit of having the meeting virtually was that it likely enabled more people to participate and could have helped overcome a number of other limitations related to stakeholder participation such as funding, work, and long-distance travel-related constraints (30).

In conclusion, the PE-CGS Network is actively pursuing a goal to promote best practices for participant engagement in cancer genome sequencing research. These efforts harness the collective strengths of all Network stakeholders. The PE-CGS Network is positioned to generate discoveries and resources that will advance the science of engagement in cancer genomics research and provide real benefits to our patients. These scientific insights offer the potential to be applied to the development of new cancer therapies, improvement of methods of cancer diagnosis, or identification of opportunities for cancer prevention. The impact likely extends beyond the PE-CGS Network as publications will be made immediately available and data from the Network will be made available to the broader scientific community in a manner consistent with the participant informed consent and community agreement.

## Supplementary Material

Banner Authorship Members Banner authorship list
