# Supplementary material for: Priorities to Promote Participant Engagement in the Participant Engagement and Cancer Genome Sequencing (PE-CGS) Network
Source: Cancer Epidemiol Biomarkers Prev. 2023 Feb 15;32(4):487–95. doi: 10.1158/1055-9965.EPI-22-0356 (PMC10068438; doi:10.1158/1055-9965.EPI-22-0356)
Supplement: Banner Authorship Members — Banner authorship list [file epi-22-0356_banner_authorship_members_suppsm.docx]

| **NCI/NIH** |
| --- |
| Kelly. K. Filipski, Elizabeth M Gillanders, Rachel Hanisch, Leah Mechanic, Jessica Tiner, Sylvia Chou Wen-Ying |
|  |
| **OSU Coordinating Center** |
| John FP Bridges, Norah L Crossnohere, Heather Hampel, Qin Ma, Electra Paskett, Anne Schuster |
|  |
| **Count Me In** |
| Saud Al Dubayan, Elana Anastasio, Megan Anderson, Sidney Benich, Saveliy Belkin, Sabrina Camp, Evelina Ceca, Claudia Chu, Alanna Church, Carrie Cibulskis, Brian Crompton, Taylor Cusher, Lorena De La Vega, George Demetri, Lauren Fisher, Stacey Gabriel, Judy Garber, Suzanne George, Gad Getz, Katherine Janeway, Kristen Kubler, Elizaveta Leshchiner, Jennifer Mack, Adrian Marino-Enriquez, Ashley Matthews, Colleen Nguyen, Corrie Painter, Chandrajit Raut, Brendan Reardon, Tim Rebbeck, Kristy Schlueter-Kuck, Jill Stopfer, Eliezer Van Allen, Andrew Khalaj, Nikhil Wagle, Alex Wilson, Sarah Winnicki |
|  |
| **Engagement of American Indians of Southwestern Tribal Nations in Cancer Genome Sequencing** |
| Shoshana Adler-Jaffe, Leslie Andritsos, Lorenda Belone, Cindy Blair, Tawny Boyce, Cory Broehm, Ursa Brown-Glaberman, Sara Byron, Hershel Clark, Linda Cook, Zoneddy Dayao, Erin Fitzgerald, Melissa Gonzales, Stacy Gray, Yan Guo, Joshua Hanson, Jeffrey Henderson, Patricia Nez Henderson, Dakota Jim, Nancy Joste, Huining Kang, Miria Kano, Jonathan Keats, Mikaela Kosich, Richard Lauer, Johnnye Lewis, James Lowey, Jeff MacKeigan, Debra MacKenzie, Dennis McCance, Angela Meisner, Shiraz Mishra, Carolyn Muller, Scott Ness, Mary Oyebamiji, RaeAnn Paden, V. Shane Pankratz, Valerie Parks, Mallery Quetawki, Joseph Rodman, Shawnia Ryan, Nicholas Schork, Audrey Simplicio, Christine Serway, Andrew Sussman, Allan Tomkinson, Jeffrey Trent, Timothy Whitsett, Charles Wiggins, Cheryl Willman, Guo Yan, Jain Zhou |
|  |
| **USC Center for Optimization of Participant Engagement for Cancer Characterization (COPECC)** |
| Sandra Algaze, John Carpten, Lourdes Baez Conde, David Craig, Julie Culver, Grace Diaz, James W. Gauderman, Daisy Hernandez, Michael Hochman, Syma Iqbal, Heinz-Josef Lenz, Caryn Lerman, Juan Pablo Lewinger, Asley Noriega, Serina Ovalle, Charite N. Ricker, Crystal Rubalcava, Bodour Salhia, Ira Shulman, Mariana Stern, Jennifer Tsui, Joseph Vega, Enrique Velazquez Villarreal, Dean Wallace |
|  |
| **OPTimIzing engageMent in discovery of molecular evolution of low grade glioma (OPTIMUM)** |
| Mark Adams, Elizabeth Claus, Marilyn Coors, Matthew W. DeCamp, Catherine DesRoches, Rose Du, Kevin Hall, Brooke Dorsey Holliman, Liangge Hsu, Kevin C. Johnson, Kevin Kelly, Harlon Krumholz, Bethany Kwan, Gregory Omerza, Laura L Pyle, Jenna E Reno, Jens Rueter, Liz Salmi, Roel Verhaak, Xinming Zhuo |
|  |
| **Washington University Participant Engagement and Cancer Genomic Sequencing Center (WU-PE-CGS)** |
| Melinda Bachini, Yin Cao, Aadel Chaudhuri, Feng Chen, Graham A. Colditz, Kia L. Davis, Patricia I. Dickson, Li Ding, Bettina Drake, Eric J. Duncavage, Mark A. Fiala, Ryan C. Fields, Aimee S. James, Albert M. Lai, Kian H. Lim, Erin Lynn Linnenbringer, Christine M. Marx, Jessica Mozersky, Mary C. Politi, David Spencer, Carrie Stoll, Adetunji T. Toriola, Ravi Vij, Fei Wan, Mark A. Watson, Michael C. Wendl |
